# Stable nuclear transformation of *Pandorina morum*

**DOI:** 10.1186/1472-6750-14-65

**Published:** 2014-07-17

**Authors:** Kai Lerche, Armin Hallmann

**Affiliations:** 1 Department of Cellular and Developmental Biology of Plants, University of Bielefeld, Universitätsstr. 25, D-33615 Bielefeld, Germany

**Keywords:** Co-transformation, *Gaussia princeps* luciferase gene, Genetic engineering, Green algae, Heterologous expression, Reporter genes, Selectable markers, *Streptomyces rimosus aph*VIII gene, Volvocaceae, Volvocine algae

## Abstract

**Background:**

Volvocine green algae like *Pandorina morum* represent one of the most recent inventions of multicellularity diverged from their unicellular relatives. The 8–16 celled *P. morum* alga and its close multicellular relatives constitute a model lineage for research into cellular differentiation, morphogenesis and epithelial folding, sexual reproduction and evolution of multicellularity. *Pandorina* is the largest and most complex organism in the volvocine lineage that still exhibits isogamous sexual reproduction. So far, molecular-biological investigations in *P. morum* were constricted due to the absence of methods for transformation of this species, which is a prerequisite for introduction of reporter genes and (modified) genes of interest.

**Results:**

Stable nuclear transformation of *P. morum* was achieved using chimeric constructs with a selectable marker, a reporter gene, promoters and upstream and downstream flanking sequences from heterologous sources. DNA was introduced into the cells by particle bombardment with plasmid-coated gold particles. The aminoglycoside 3′-phosphotransferase VIII (*aph*VIII) gene of *Streptomyces rimosus* under control of an artificial, heterologous promoter was used as the selectable marker. The artificial promoter contained a tandem arrangement of the promoter of both the heat shock protein 70A (*hsp*70A) and the ribulose-1,5-bisphosphat-carboxylase/-oxygenase S3 (*rbc*S3) gene of *Volvox carteri*. Due to the expression of *aph*VIII, transformants gained up to 333-fold higher resistance to paromomycin in comparison to the parent wild-type strain.

The heterologous luciferase (*gluc*) gene of *Gaussia princeps*, which was previously genetically engineered to match the nuclear codon usage of *Chlamydomonas reinhardtii*, was used as a co-transformed, unselectable reporter gene. The expression of the co-bombarded *gluc* gene in transformants and the induction of *gluc* by heat shock were demonstrated through bioluminescence assays.

**Conclusion:**

Stable nuclear transformation of *P. morum* using the particle bombardment technique is now feasible. Functional expression of heterologous genes is achieved using heterologous flanking sequences from *Volvox carteri* and *Chlamydomonas reinhardtii*. The *aph*VIII gene of the actinobacterium *S. rimosus* can be used as a selectable marker for transformation experiments in the green alga *P. morum*. The *gluc* gene of the marine copepod *G. princeps*, expressed under control of heterologous promoter elements, represents a suitable reporter gene for monitoring gene expression or for other applications in *P. morum*.

## Background

**Figure 1:**
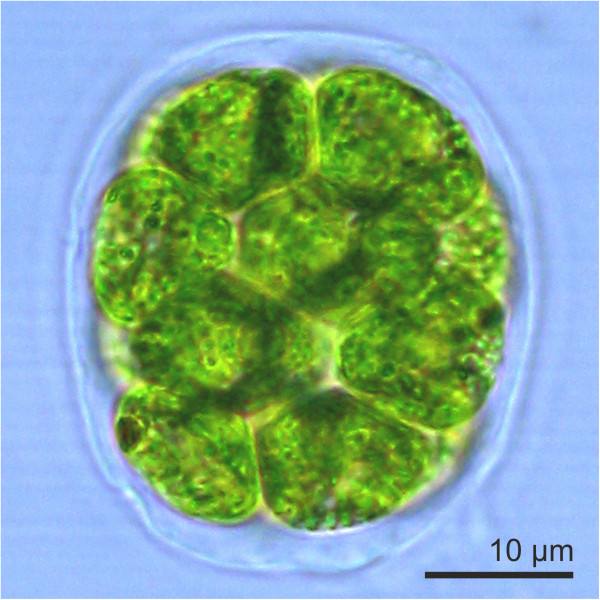
**The wild-type phenotype of *P. morum.*** A vegetatively grown, ellipsoid spheroid of *P. morum* with ~16 cells. The cells are close together and even slightly narrowed at the inner end to appear keystone- or pear-shaped. Each cell has two flagella with two contractile vacuoles at their base, a single massive, cup-shaped chloroplast with at least one pyrenoid, and an eyespot. The cells are surrounded by a complex but transparent glycoprotein-rich extracellular matrix that holds all of the cells in place.

The volvocine green algae, a group of ~50 species involving the families Chlamydomonaceae, Goniaceae and Volvocaceae [1], serve as a model lineage for research into cellular differentiation, morphogenesis, epithelial folding and evolution of multicellularity [2-4]. Furthermore, this group is used to study the evolution of sexual reproduction, including the evolution of anisogamy [2,5]. In many lineages, like higher plants and animals, the evolution of multicellularity and sexual reproduction occurred way back in evolutionary history and the molecular origins therefore are obscured by genetic drift. In the volvocine algae the development from unicellular species, which were similar to the present species *Chlamydomonas reinhardtii*[5], to multicellular species and its relatives occurred only about 200 million years ago [1]. Well-known multicellular species in this group are *Volvox*, *Pleodorina*, *Eudorina*, *Pandorina* and *Gonium*[2,4]. The 8–16 celled *Pandorina morum* (Figure 1) [6,7] is intermediate in organizational complexity between the unicellular alga *C. reinhardtii* and the multicellular alga *Volvox carteri* (2,000–4,000 cells) and it has not been subject to molecular analyses and genetic engineering due to a lack of biotechnological possibilities.

The cells of *P. morum* form an ellipsoid spheroid with a distinct anterior-posterior polarity. The way of vegetative reproduction and mode of daughter colony formation where the young colonies break out of the old parental layers reminded Bory of the legend of Pandora’s box; thus, he applied the French diminutive, *Pandorina*, to the genus he named [7]. There are several distinctive features of *Pandorina* that warrant its inclusion in the series of volvocine algae to be analyzed molecularly in the future. *Pandorina* has a special morphology among the volvocine algae: the cells fill nearly the entire volume of the colony, as opposed to other, larger volvocine algae in which the cells are arranged at the periphery of the colony, with extracellular matrix filling the space between cells. The compact morphology characteristic of *Pandorina* (Figure 1) appears to be derived from ancestors with expanded ECM [8]. Furthermore, *Pandorina* is the smallest volvocine alga to exhibit a ‘colonial boundary’, the tripartite boundary that surrounds the entire colony. This ‘colonial boundary’ is the most conserved morphological feature of the Volvocalean extracellular matrix [9]. *Pandorina* is moreover the largest and most complex organism in the volvocine lineage that still exhibits isogamous sexual reproduction [2,4]. Thus, the gametes of *P. morum* are almost identical in morphology, differing genetically only in allele expression in the mating-type locus (mating type plus or minus) [10]. The molecular-biological investigation and the comparison of molecular characteristics between the isogamous sexual reproduction in *P. morum* and anisogamous species like *Eudorina* could reveal insights into the evolution of anisogamy, but such studies are hampered by the absence of methods for genetic engineering of *P. morum*. In contrast, the anisogamous species *Eudorina* is accessible for genetic manipulations [11]. The technical feasibility of genetically manipulating a species of interest is a key factor for any detailed molecular analysis of gene or protein functions.

The nuclear genome of *P. morum* has not been sequenced, only its mitochondrial genome has been revealed [12]. Actually, only the genomes of *C. reinhardtii*[13] and *V. carteri*[14] have been sequenced in the group of volvocine algae. For both species effective transformation protocols exist and they are accessible to molecular manipulations [15-19]. Nuclear transformation protocols also exist for the volvocine algae *Gonium pectorale*[20] and *Eudorina elegans*[11]. There is a single transformation technique that worked in all of the previously transformed volvocine species, namely particle bombardment.

Aside from the nuclear transformation protocol, selectable markers and reporter genes are required for genetic manipulations. In the volvocine algae, selectable markers and reporter genes have mainly been established for *C. reinhardtii*[5,21-28] and, to a lesser extent, for *V. carteri*[28-31], *G. pectorale*[20] and *E. elegans*[11]. In the present study, we wanted to check whether the vectors and transformation methods that were developed earlier for volvocine algae — in particular those for *G. pectorale*[20] and *E. elegans*[11] — can be applied with equal success to *Pandorina*.

The most promising candidate for a selectable marker gene in *Pandorina* was the *aph*VIII gene of *Streptomyces rimosus* that has been used as a selectable marker in *C. reinhardtii*[25], *V. carteri*[30,31], *G. pectorale*[20] and *E. elegans*[11]. The *aph*VIII gene codes for an aminoglycoside 3′-phosphotransferase enzyme that catalyzes the transfer of the γ-phosphate of ATP to the 3′-position of an aminoglycoside antibiotic like paromomycin; this transfer chemically modifies and inactivates the antibiotic molecule. Paromomycin otherwise inhibits cell growth by blocking the protein synthesis of ribosomes at the translocation stage [32].

The best candidate for a useful reporter gene in *P. morum* seemed to be the luciferase gene (*gluc*) of the deep sea copepod *Gaussia princeps*[33,34], which was previously adapted to the nuclear codon usage of *C. reinhardtii*[27]. The Gluc protein catalyzes the oxidation of the substrate coelenterazine into the excited form of coelenteramide that releases a photon with a wavelength of 480 nm upon electron transition to the ground state [34]. The blue light produced by this reaction can be detected by various methods like luminometer measurements and assays on light sensitive films [35]. The *gluc* reporter gene has successfully been introduced into *C. reinhardtii*[27], *G. pectorale*[20] and *E. elegans*[11] and it was functionally expressed in all three organisms.

The selectable markers and reporter genes also require appropriate promoters. Based on previous results, promising candidates were the *C. reinhardtii* promoter of the abundant protein of photosystem I complex gene (*PSAD*) [36], the *C. reinhardtii* promoter of the heat shock protein 70A (*HSP70A*) [37], and the *V. carteri* tandem promoter of the *hsp*70A and *rbc*S3 genes [31]. The *C. reinhardtii HSP70A* promoter and the *V. carteri hsp*70A/*rbc*S3 tandem promoter have already proved useful for expression of the *aph*VIII gene in *V. carteri*, *C. reinhardtii*, *G. pectorale* and *E. elegans*[11,20,30].

Here we demonstrate the stable nuclear transformation of *P. morum* using chimeric constructs containing heterologous *aph*VIII and *gluc* genes under the control of heterologous 5′ and 3′ flanking sequences from *V. carteri* and *C. reinhardtii*. The enzyme activity of the heterologously expressed *Gaussia* luciferase was detectable by luminometer measurements and bioluminescence assays. The expression of the heterologous *gluc* gene was further increased in transformants containing the *gluc* gene under control of the heterologous tandem promoter including the heat shock promoter, if the transformants were subject to a temporary shift to higher temperatures.

## Results

### Antibiotic tolerance of wild-type *Pandorina* algae

**Figure 2:**
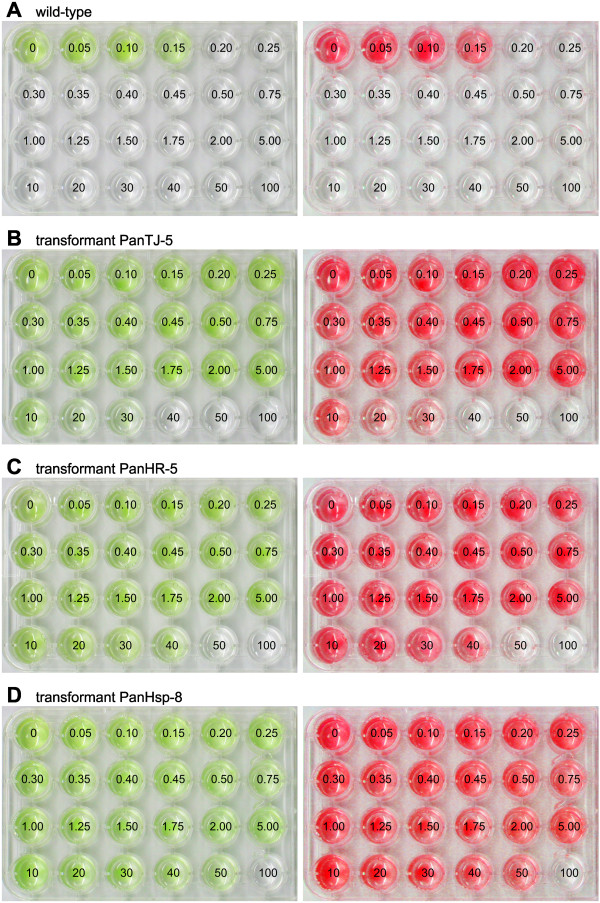
**Analysis of paromomycin resistance in wild-type and transgenic *P. morum *strains.** For detailed analysis of resistance, identical quantities of *P. morum* cells were exposed to increasing concentrations of paromomycin, and incubated for 10 days. Numbers refer to the concentration of paromomycin [μg/ml] utilized. Natural color (left) and red-shifted, false-color images (right) are shown. **(A)** The wild-type *P. morum* strain. **(B-D)** Transgenic *P. morum* strains co-transformed with pPmr3, the selectable marker plasmid, in addition to a second, non-selectable reporter gene plasmid. **(B)** Transformant PanTJ-5 was co-transformed with the plasmids pPmr3 and pPsaD-GLuc. **(C)** Transformant PanHR-5 was co-transformed with the plasmids pPmr3 and pHRLucP. **(D)** Transformant PanHsp-8 was co-transformed with the plasmids pPmr3 and pHsp70A-GLuc.

To allow for selection of transformants with even weak transgene-mediated resistance, the lowest concentration of the antibiotic paromomycin that kills all wild-type *Pandorina* cells was determined. To achieve this, a series of aliquots with the same number of wild-type *P. morum* cells was exposed to increasing concentrations of paromomycin, incubated for 10 days, and screened for living (green) or dead (white) cells. The alga *P. morum* tolerated concentrations of up to 0.15 μg/ml paromomycin. A concentration of 0.20 μg paromomycin/ml or higher led to 100% cell death (Figure 2A). To allow for an objective and rapid discrimination between aliquots containing living cells and aliquots containing dead cells even in large-scale screenings of culture plates, red-shifted false-color images were created from standard photographs of the plates (Figure 2A, right panel). Red-shifting makes living cells much easier to distinguish from dead cells.

### Transformation experiments using *aph*VIII as a selectable marker

The establishment and optimization of the transformation protocol was done by changing several parameters systematically (Additional file 1). In the most successful protocol, 150 ml of a logarithmically growing *P. morum* culture with a density of 7 × 10^4^ cells/ml was harvested by centrifugation. The concentrated algae were spread and immobilized on a cellulose acetate membrane filter and subjected to particle bombardment. The total number of cells used for each transformation experiment was about 9 × 10^6^. Different settings of the Biolistic PDS-1000/He (Bio-Rad) particle delivery system were tested to obtain the maximum number of paromomycin resistant transformants (Additional file 1). The most effective protocol for the biolistic transformation of *P. morum* using DNA-coated gold microprojectiles is described in the Materials and Methods section. The parameters for the most effective transformation protocol are summarized in Table 1.

**Table 1:** **Most successful combination of parameters for *P. morum *** **transformation using a PDS-1000/He biolistic device**

**Parameter**	**Parameter specification**
Material of microprojectiles	gold
Size of microprojectiles	0.6 μm in diameter
Selectable marker plasmid	pPmr3
Coating of microprojectiles	plasmid-DNA/microcarrier/CaCl_2_/spermidine/ ethanol -precipitation
Target cells	immobilized on cellulose acetate membrane filter; almost free of liquid
Burst pressure of rupture disk	1350 psi
Rupture disk-macrocarrier distance	7 mm
Macrocarrier-stopping screen distance	8 mm
Stopping screen-target cell distance	6 cm
Chamber evacuation	27 inch Hg
Cultivation after particle bombardment	in liquid medium

**Figure 3:**
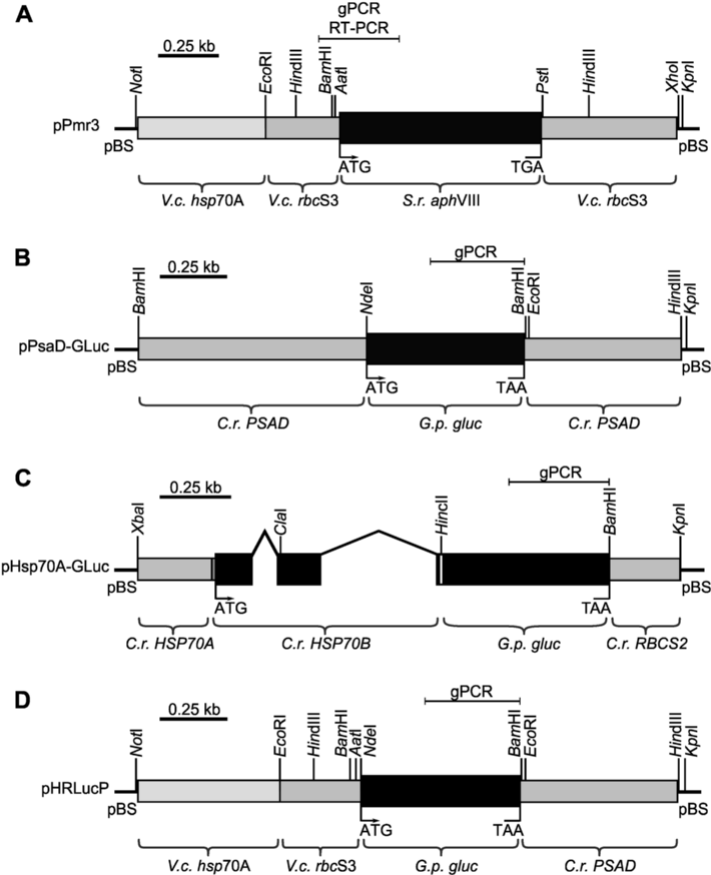
**Schematic diagram of the selectable marker plasmid and co-transformed, non-selectable plasmids. (A)** The chimeric selectable marker plasmid pPmr3. **(B-D)** Co-transformed, non-selectable plasmids containing reporter genes: **(B)** pPsaD-GLuc, **(C)** pHsp70A-GLuc, and **(D)** pHRLucP. The angled lines in (C) indicate introns. (A-D) Amplified genomic (gPCR) or RT-PCR fragments are indicated. *V.c.*, *Volvox carteri*; *C.r.*, *Chlamydomonas reinhardtii*; *S.r.*, *Streptomyces rimosus*; *G.p.*, *Gaussia princeps*; *gluc*, luciferase gene; pBS, pBluescript II vector.

For all transformation experiments, a chimeric, *aph*VIII-based plasmid (pPmr3) [31] was used as a selectable marker that confers resistance to paromomycin. The pPmr3 plasmid contains the coding sequence of the *Streptomyces rimosus aph*VIII gene, a 5′-flanking sequence that includes an artificial tandem promoter from the *hsp*70A and *rbc*S3 genes of *V. carteri*, and a 3′-flanking sequence derived from the *rbc*S3 gene of *V. carteri* (Figure 3A) [11,20].

Unselectable plasmids were co-transformed with the selectable marker plasmid pPmr3. For use as a reporter gene, the *gluc* gene was brought under control of different heterologous 5′ and 3′ flanking sequences from *V. carteri* and *C. reinhardtii*. The utilized plasmids were pPsaD-GLuc, pHsp70A-GLuc or pHRLucP (Figure 3B-D) [11,20]. After the transformation procedure, the algae were incubated in liquid medium and allowed to recover for 24 h before paromomycin was added. Within 48 h all non-transformed algae died, which resulted in a temporary clarification of the medium. After 10–14 days of incubation, re-greening of the incubation flasks indicated the initial presence and reproduction of at least one paromomycin-resistant transformant per flask. Using this protocol, a total of 38 paromomycin resistant clones were obtained in 4 independent transformation experiments.

### Paromomycin resistance of transformants

The antibiotic resistance of the 38 transformants was justified and concretized by incubation for 10 days in 1 ml JM medium with different concentrations of paromomycin ranging from 0.05 to 100 μg/ml (for concentrations see Figure 2). Transformants with the most robust resistance tolerated concentrations up to 50 μg/ml paromomycin (Figure 2B-D, Table 2). Thus, transformants of *P. morum* generated with the selectable marker plasmid pPmr3 exhibit an up to 333-fold higher tolerance to paromomycin than the wild-type strains. The variation in resistance between different clones is quite common in transformation experiments [11,20]. This could be the result of gene-dosage effects caused by variable number of copies integrated into the genome and/or it can reflect position effects on expression of the transgene due to its random integration into the genome.

**Table 2:** Results of paromomycin resistance assays*

**Transformant**	**Resistance up to x μg/ml paromomycin**	**Transformant**	**Resistance up to x μg/ml paromomycin**	**Transformant**	**Resistance up to x μg/ml paromomycin**
PanTJ-1	50	PanHR-1	50	PanHsp-1	40
PanTJ-2	50	PanHR-2	50	PanHsp-2	50
PanTJ-3	50	PanHR-3	40	PanHsp-3	50
PanTJ-4	50	PanHR-4	50	PanHsp-4	50
PanTJ-5	30	PanHR-5	40	PanHsp-5	50
PanTJ-6	40	PanHR-6	30	PanHsp-6	40
PanTJ-7	50	PanHR-7	40	PanHsp-7	40
PanTJ-8	40	PanHR-8	40	PanHsp-8	50
PanTJ-9	40	PanHR-9	40	PanHsp-9	50
PanTJ-10	40	PanHR-10	40	PanHsp-10	50
		PanHR-11	50	PanHsp-11	50
		PanHR-12	50	PanHsp-12	40
		PanHR-13	50		
		PanHR-15	50		
		PanHR-21	40		
		PanHR-22	40		

*tolerance of the wild-type strain *P. morum* SAG 32.96: 0.15 μg/ml paromomycin.

### Stable genomic integration and expression of the artificial *aph*VIII gene

**Figure 4:**
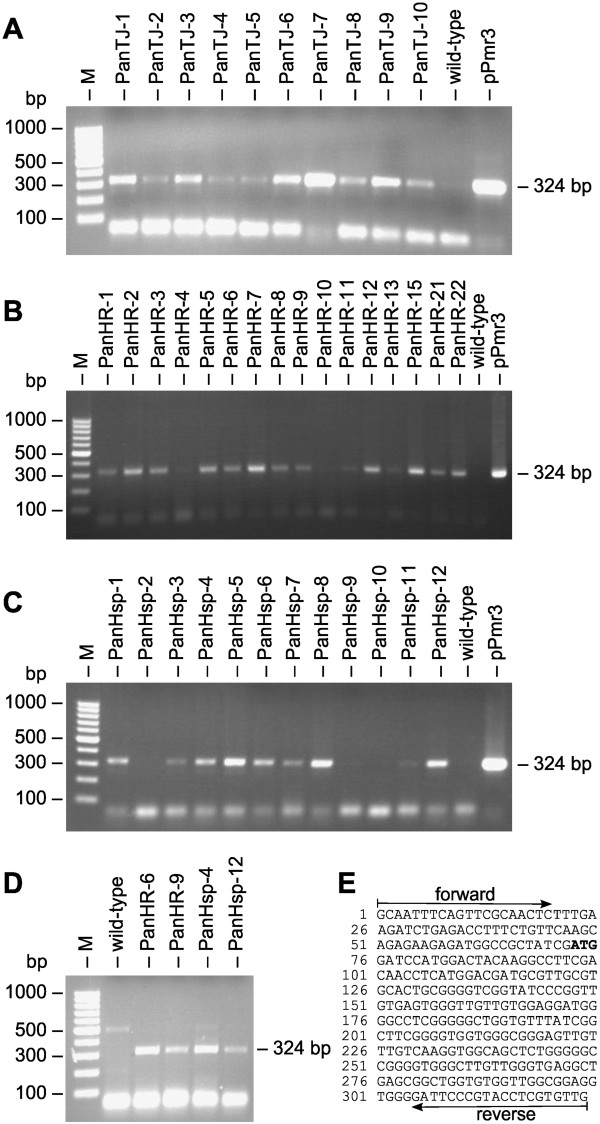
**Detection of the *aph*VIII gene in transformants and demonstration of its transcription. (A-C)** Paromomycin resistant transformants and the parental wild-type strain were analyzed for presence of the *aph*VIII gene in the genome by genomic PCR. The expected size of the PCR fragment produced in transformants was 324 bp (Figure 3A). The rightmost lane (pPmr3) shows a positive control using pPmr3 plasmid DNA as the template. Lane M refers to the molecular weight marker. **(D)** RT-PCR analysis of *aph*VIII expression. RNA from paromomycin-resistant transformants was reverse transcribed and products were amplified by PCR using primers specific for the *aph*VIII gene construct. Transformants with *aph*VIII expression were expected to yield a 324-bp cDNA fragment (Figure 3A). The parent wild-type strain was used as a control. Lane M, molecular weight marker. **(E)** Sequence obtained from the amplified and cloned genomic and cDNA fragments. The 324-bp fragment contains part of the *aph*VIII gene and part of the 5′ untranslated region from *V. carteri rbc*S3. The positions of the primers and the start codon (bold) are indicated.

PCR was used to verify the stable integration of the pPmr3 vector with the artificial *aph*VIII gene into the genome of *P. morum* transformants. The artificial *aph*VIII gene was driven by the *V. carteri hsp*70A/*rbc*S3 tandem promoter and it confers resistance to paromomycin. Genomic DNA was isolated from the 38 transformants about 100 generations after the transformants had been generated. The isolated DNA was used as a template for PCR and a 324 bp chimeric fragment of genomic DNA, containing 107 bp of the *rbc*S3 promoter region and 217 bp of the *aph*VIII coding region, was expected to be amplified with the corresponding specific primers. PCR fragments of the expected size were obtained from many clones (Figure 4A-C); the fragments were cloned and sequenced (Figure 4E). In Figures 4B and 4C the lanes for the transformants PanHR-4, PanHR-10, PanHsp-2, PanHsp-9, PanHsp-10 and PanHsp-11 show no or only weak bands, which might reflect disturbance of the PCR reaction caused by too small DNA amounts, impurities of the genomic DNA or other reasons. Anyway, the repetition of the PCRs with fresh DNA isolates from these six clones yielded fragments of the expected size (data not shown). Moreover, growth of all 38 transformants in the presence of antibiotic already demonstrated the functional expression of the *aph*VIII gene (Figure 2, Table 2).

In addition to the provided indirect evidence of *aph*VIII expression through the mediated antibiotic resistance in transformants, the transcription of the *aph*VIII gene was also examined directly by RT-PCR analysis. For it, RNA from paromomycin-resistant transformants was reverse transcribed, and products were amplified by PCR using *aph*VIII specific primers. A cDNA fragment with 324 bp was expected. Actually, a PCR fragment of the expected size was obtained from all investigated paromomycin-resistant transformants, but not from the wild-type (Figure 4D). The amplified fragments were cloned and sequenced (Figure 4E).

### Calculation of transformation frequency

The transformation frequency was calculated as the ratio of transformants that exhibited both resistance against paromomycin and the correct *aph*VIII fragment following PCR and the total number of cells used for each transformation experiment (9 × 10^6^ cells). Based on the results of 4 independent transformation experiments, a transformation frequency of 1.2 × 10^-6^ per cell was calculated. This corresponds to a transformation frequency of about 1.9 × 10^-5^ per organism.

### Plasmids for co-transformation with non-selectable reporter gene constructs

To investigate the efficiency of co-transformation in *P. morum,* three different plasmids were used containing the *gluc* gene of *G. princeps*, which was previously engineered to match the codon usage of *C. reinhardtii*[27].

Plasmid pPsaD-GLuc contains the *gluc* gene under control of 5′ and 3′ control elements of the *PSAD* gene of *C. reinhardtii*[27,36] (Figure 3B). In three volvocine species, i.e., *C. reinhardtii, G. pectorale* and *E. elegans*, the *PSAD* promoter was shown to mediate strong expression [11,20,27].

Plasmid pHsp70A-GLuc [11,20,27] (Figure 3C) contains the *gluc* gene flanked by the *C. reinhardtii HSP70A* 5′UTR and the *RBCS2* 3′UTR of *C. reinhardtii*. The *HSP70A* promoter was shown to mediate not only strong but also heat-inducible expression. The plasmid also contains the first three exons and two introns of the *C. reinhardtii HSP70B* gene that codes for the chloroplast transit peptide of the HSP70B protein [27,38,39].

Except for flanking sequences of *C. reinhardtii*, we also tested flanking sequences of *V. carteri.* Plasmid pHRLucP contains the coding region of the *gluc* gene fused to a *V. carteri hsp*70A/*rbc*S3 tandem promoter and a *C. reinhardtii PSAD* 3′UTR (Figure 3D). Like in plasmid pHsp70A-GLuc, the tandem promoter should mediate not only strong but also heat-inducible expression.

### Stable integration of non-selectable reporter gene constructs into the genome

**Figure 5:**
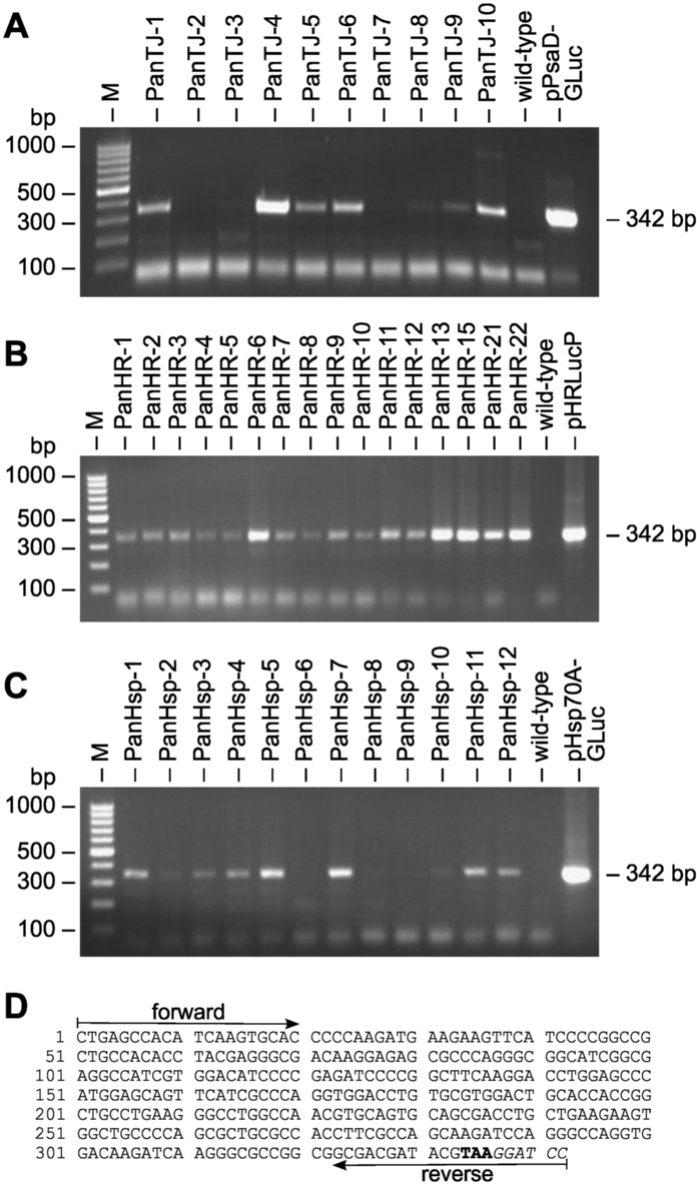
**Demonstration of co-transformation of heterologous genes.** Paromomycin-resistant transformants were analyzed for the presence of the co-transformed, non-selectable DNA in the genome. PCRs were conducted using genomic DNA from transformants co-transformed with the pPsaD-GLuc **(A)**, pHRLucP **(B)** or pHsp70A-GLuc **(C)** plasmids as template. The parental wild-type strain was analyzed as a control. Primers were specific for the heterologous *gluc* gene, and a 342-bp PCR fragment (Figure 3B-D) was expected in co-transformants. The rightmost lane shows a positive control using DNA of the co-transformed plasmid as the template. Lane M refers to the molecular weight marker. **(D)** Sequence obtained from the amplified and cloned *gluc* fragments. Primer positions, the stop codon (bold), and a *Bam*HI restriction site (italics) are indicated.

Transformants were generated by co-transformation with the selectable pPmr3 plasmid and one of the three *gluc*-derived, non-selectable reporter gene plasmids at a time. The obtained transformants were screened for stable integration of the reporter gene constructs by PCR. For it, a PCR reaction was performed using genomic DNA of transformants as a template and *gluc*-specific oligonucleotides. The expected fragment was 342 bp in size. Such a fragment was obtained from transformants produced by co-transformation with the plasmids pPsaD-GLuc (7 positive transformants), pHRLucP (16 positive transformants) and pHsp70A-GLuc (10 positive transformants) (Figure 5A-C). As expected, PCRs with genomic DNA of the wild-type yielded no such product. The identity of the amplified fragments was verified by cloning and sequencing (Figure 5D). Based on the results of the PCR experiments, the co-transformation rate in *P. morum* was determined to be 84% (±15%). The co-transformation rate was defined as the rate of transformants containing the *gluc* gene among the transformants containing the *aph*VIII gene.

### Functional expression of co-transformed reporter genes

**Figure 6:**
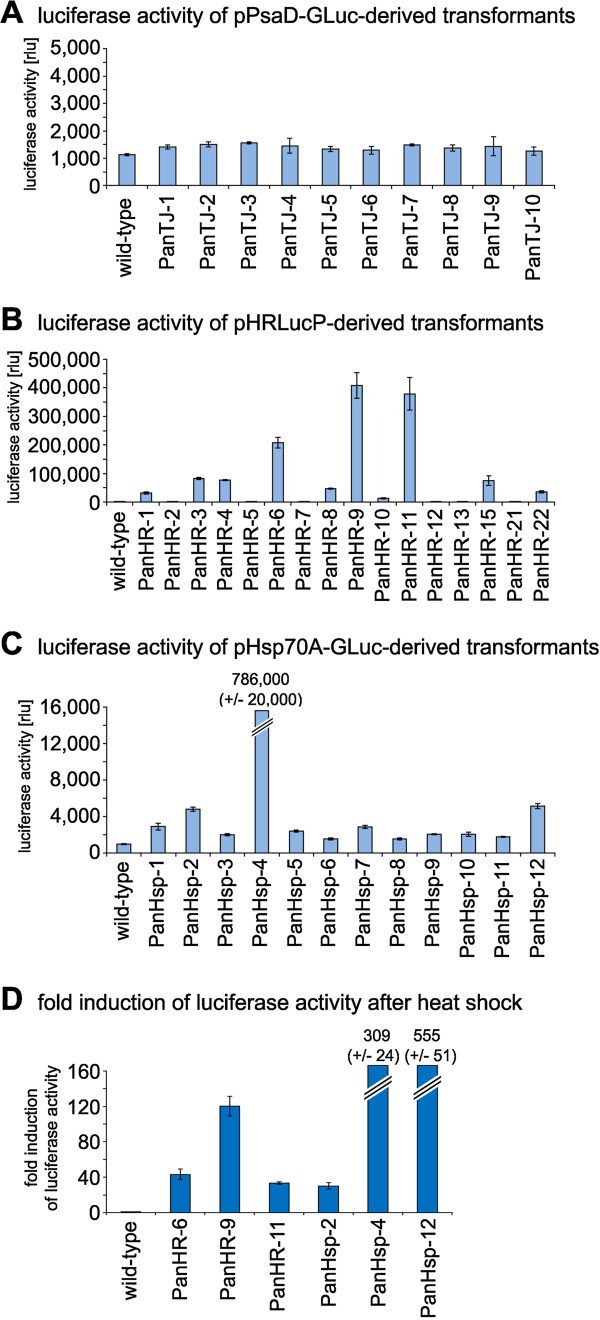
**Quantification and inducibility of luciferase activity in transformants expressing the luciferase gene.** Luciferase activity in transformants was assayed using a luminometer. The bars represent the mean of three independent experiments. The standard deviation is indicated. **(A)** Luciferase activity of pPsaD-GLuc derived transformants (PanTJ…) compared to the wild-type strain. **(B)** Luciferase activity of pHRLucP derived transformants (PanHR…) compared to the wild-type strain. **(C)** Luciferase activity of pHsp70A-GLuc derived transformants (PanHsp…) compared to the wild-type strain. **(D)** Fold induction of luciferase activity in heat-shocked transformants (39°C) compared to non-heat-shocked transformants. In pHRLucP-derived transformants (PanHR…) the luciferase is driven by the *V. carteri hsp*70A/*rbc*S3 tandem promoter. For pHsp70A-GLuc-derived transformants (PanHsp…) the luciferase gene is driven by the *C. reinhardtii HSP70A* promoter. The parental wild-type strain was analyzed as a control. Transformants and wild-type colonies were subjected to a temperature shift from 29°C to 39°C for 1 h. After a 15 min recovery phase at 29°C, cells were lysed, and luciferase activity was assayed. As a reference control, non-heat-shocked transformants were analyzed in an identical fashion.

Transformants generated by co-transformation with the selectable marker plasmid pPmr3 and the *gluc-*derived plasmids pPsaD-GLuc, pHRLucP and pHsp70A-GLuc were assayed for functional expression of luciferase. For it, cell extracts of all transformants were checked for luciferase activity using a luminometer (Figure 6A-C).For some reason, no significant luciferase activity was observed in transformants generated with pPsaD-GLuc (Figure 6A). The relative light units (rlu) detected in these transformants are about the same as in wild-type cells and correspond to the background light, which is at about 1,000 rlu.

In contrast, luciferase activity was detected in several transformants generated by co-transformation with the plasmid pHRLucP (Figure 6B). In this DNA construct the *gluc* gene is under control of the artificial tandem promoter from the *hsp*70A and *rbc*S3 genes of *V. carteri*. Extracts of ten out of sixteen transformants clearly showed emission of light and, thus, functional expression of luciferase. The amount of detected relative light units (rlu) is significantly higher than in the wild-type strain (853 ± 11 rlu). The transformants PanHR-9 and PanHR-11 exhibited the highest Gluc activity with 407,500 ± 45,000 rlu and 378,500 ± 57,000 rlu, respectively.

Extracts of transformants generated by co-transformation with the plasmid pHsp70A-GLuc showed a significant but mostly moderate luciferase activity when cultivated under standard conditions at 29°C (Figure 6C). In this DNA construct the *gluc* gene is under the control of the *C. reinhardtii HSP70A* promoter; there is also the genomic sequence of the *C. reinhardtii HSP70B* chloroplast transit peptide containing two introns that precedes the *gluc* coding sequence. Only transformant PanHsp-4 exhibited a high Gluc activity of 786,000 ± 20,000 rlu under these conditions, while the wild-type strain showed only 966 ± 50 rlu.

The DNA constructs in pHRLucP and pHsp70A-GLuc are driven by heat-shock promoters that show inducibility by heat in their donor organisms *V. carteri* and *C. reinhardtii*, respectively.

**Figure 7:**
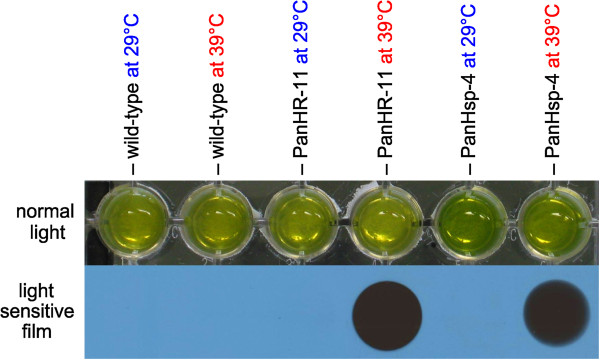
**Visualization of inducible luciferase activity.** Luciferase assay results for parental wild-type colonies and for several transformants with or without heat shock. The transformants PanHR-11 and PanHsp-4 were generated with the co-bombarded plasmids pHRLucP and pHsp70A-GLuc, respectively. Algae cultures were divided into two aliquots; one aliquot was subjected to a heat shock at 39°C for 1 h, and the other aliquot was maintained at 29°C. Upper row: standard photo showing the assay setup (normal light). Lower row: exposure to a light-sensitive film.

Analysis for inducibility of these promoter elements in *P. morum* was done by subjecting the algae transformed with the plasmids pHRLucP and pHsp70A-GLuc to a temperature shift. The temperature was changed from 29°C to 9 different temperatures between 27°C and 51°C. The strongest increase in luciferase activity was achieved by a temperature shift to 39°C for 1 h. In extracts of transformants generated by co-transformation with plasmid pHRLucP the luciferase activity increased between 33- and 120-fold compared to algae that were kept at 29°C. Extracts of transformants generated by co-transformation with plasmid pHsp70A-GLuc showed a 30- to 555-fold increase in luciferase activity (Figure 6D).The heat-inducibility of luciferase activity also was investigated by an assay using light sensitive films and intact, undisrupted cells. For it, an aliquot of the transformant cells produced with plasmid pHRLucP was subject to a temperature shift from 29°C to 39°C for 1 h; as a reference, another aliquot of the transformant cells was kept at 29°C. The aliquots were pipetted into separate wells of a 24-well microtiter plate, substrate solution was added and the plates were set down on a light sensitive film for 2 h in the dark. In heat-treated transformants, the light emission caused by luciferase activity was strong enough to turn the light sensitive film totally black (Figure 7). No signal was detectable in transformants that were not subject to the increase in temperature. As expected, both untreated and heat-treated wild-type cells also show no signal.

**Figure 8:**
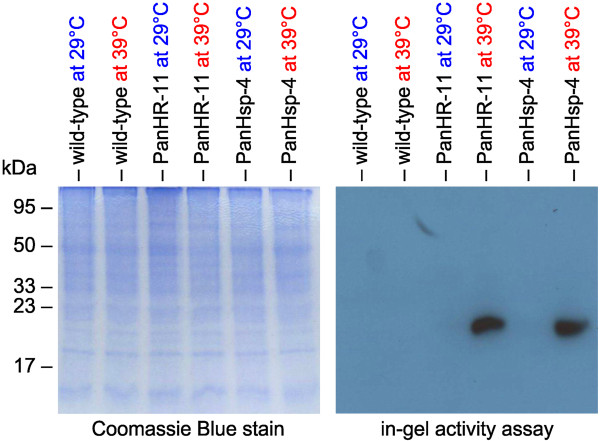
**Detection of luciferase activity by in-gel activity assays.** The transformants PanHR-11 and PanHsp-4 were generated with the co-bombarded plasmids pHRLucP and pHsp70A-GLuc, respectively. The wild-type strain was used as a control. Algae cultures were divided into two aliquots; one aliquot was subjected to a heat shock at 39°C for 1 h, and the other aliquot was maintained at 29°C. Cell extracts with equal amounts of protein were subjected to SDS-PAGE. Subsequently, in-gel renaturation was performed using β-cyclodextrin to remove SDS from the gel, the coelenterazine-substrate was added, and the gel was exposed to a light-sensitive film (right panel). As a loading control, the same extracts were also stained with Coomassie Blue following SDS-PAGE (left panel).

The molecular weight of the mature Gluc protein in *P. morum* was determined by comparison with a molecular weight marker in an in-gel activity assay. For it, cell extracts of heat induced transformants and controls were separated on a standard SDS-PAGE without thiol reagents. After electrophoresis, SDS was removed from the gel to allow for refolding of the luciferase enzyme into its active conformation. After addition of the substrate solution, the gel was set down on a light sensitive film for 2 h in the dark.In cell extracts from heat-treated transformants, generated by co-transformation with plasmid pHRLucP (Figure 8, transformant PanHR-11), light emission produced a band with a size of about 20 kDa that reflects the molecular weight of the Gluc protein (Figure 8). For transformants generated by co-transformation with plasmid pHsp70A-GLuc, a size of 31 kDa was calculated for the intact, unprocessed HSP70B/Gluc fusion protein. The in-gel activity assay loaded with cell extracts from heat-treated transformants (Figure 8, transformant PanHsp-4) shows a band of only about 20 kDa. This size reveals that the HSP70B chloroplast transit peptide has been cleaved off as expected.

The results from the in-gel activity assays substantiate the heat-inducibility of heat-shock promoters from heterologous sources in *P. morum*.

### Long term stability of DNA integration and gene expression

For any biotechnological approach with transgenic organisms, the stability of the transgenes is of fundamental importance. Therefore, the stability and expression of the *aph*VIII and *gluc* genes introduced into *P. morum* transformants was investigated after twelve months of cultivation or, in other words, after about 300 generations. Cultivation was without selective pressure during the twelve months. The presence of the heterologous genes was rechecked by PCR and the functionality of the expression products was reexamined by paromomycin resistance assays and luminometer assays. In all transformants, PCR fragments of the expected sizes were amplified and, thus, the genomic integration of the artificial gene constructs could be confirmed by PCR. All transformants exhibited about the same antibiotic resistance compared to the values determined right after transformation; the range of fluctuations was only +/-6%. Likewise, luciferase activity in the transformants was stable. The comparison of the values after twelve months of cultivation with the values determined right after transformation gave a fluctuation range of only +/-7%. Thus, both integration and expression of the heterologous *aph*VIII and *gluc* genes are stable in *P. morum*.

## Discussion

Here we demonstrate the stable nuclear transformation of the green alga *P. morum* by particle bombardment with plasmid-coated gold particles. Our approach only uses plasmids containing chimeric constructs from heterologous sources. Particle bombardment also was efficient for transformation of *C. reinhardtii*[40], *V. carteri*[15], *G. pectorale*[20] and *E. elegans*[11]. The capability for stable genetical modification of an organism of interest is an important requirement in modern molecular biology, because this technique facilitates the identification of gene functions and allows for the introduction of additional characteristics and abilities.

### Antibiotics resistance

For stable nuclear transformation of *P. morum* the *aph*VIII gene of *S. rimosus* driven by a *V. carteri hsp*70A/*rbc*S3 tandem promoter proved to be a suitable selectable marker, even though all parts of the DNA construct came from heterologous sources. Transformants generated with the *aph*VIII construct show an up to 333-fold higher tolerance to the aminoglycoside antibiotic paromomycin compared to the parent wild-type strain. When the *aph*VIII gene was used as a selectable marker in other volvocine algae, the factor for the increase in antibiotic tolerance in transformants was similar or lower. For *V. carteri* the increase in antibiotic tolerance was about 100-fold [30,31], it was 100 to 200 fold in *C. reinhardtii*[25,30], about 330-fold in *E. elegans*[11] and 15 to 40 fold in *G. pectorale*[20]. Even if the antibiotic tolerance of transformants varies between the investigated volvocine species, the *aph*VIII gene allowed for selection of transformants in all these species.

### Transformation frequency

The frequency of transformation was calculated to be 1.2 × 10^-6^ per cell in *P. morum*. The transformation frequency in *V. carteri* was estimated to be 2.5 × 10^-5^[15]. In *C. reinhardtii* the frequency of transformation was 1.3 - 1.9 × 10^-7^[41]; it was 1.1 – 6.6 × 10^-7^ in *G. pectorale*[20] and 3.7 × 10^-7^ in *E. elegans*[11]. Thus, the transformation frequency in *P. morum* is about 20 times lower than in *V. carteri* but about 2 to 10 times higher than in *C. reinhardtii*, *G. pectorale* and *E. elegans*. On an overall basis, the transformation frequencies in volvocine algae vary by about two orders of magnitude (1.1 × 10^-7^ to 2.5 × 10^-5^). There is limited information about transformation frequencies in other algae with the exception of some diatom species. The reported frequencies are 1 × 10^-7^ - 1 × 10^-6^ per cell in *Phaeodactylum tricornutum*[42] and 3 × 10^-8^ - 3 × 10^-7^ per cell in *Cyclotella cryptica* and *Navicula saprophila*[43]. Therefore, in diatoms the frequency of transformation is similar or somewhat lower compared to that in volvocine algae.

### Co-transformation rate

Co-transformation of a selectable marker plasmid and the gene of interest on a separate plasmid is a common strategy especially when large genes and complex gene constructs have to be integrated into the genome [16,20,24,26,29,31,44-46]. Another advantage of co-transformation is that the same selectable marker plasmid can be used for all transformation experiments. In *P. morum* the co-transformation rate was calculated to be at about 80%. For *V. carteri* co-transformation rates of 10% - 60% [29] or 40% - 80% [15] have been reported. The co-transformation rate was about 80% in *C. reinhardtii*[26], 30% - 50% in *G. pectorale*[20] and 50% - 100% in *E. elegans*[11]. The co-transformation rates in volvocine algae are high in all volvocine species and particularly in *P. morum*. Therefore, transformation of *P. morum* does not require construction of vectors that contain all required genes and regulatory elements on a single plasmid.

### Expression of heterologous genes using heterologous promoters

Some promoters from *C. reinhardtii* have been shown to work in *V. carteri* and vice versa [30]. Likewise, some promoter elements from *C. reinhardtii* and *V. carteri* also work in *G. pectorale* and *E. elegans*[11,20]. Here we show for *P. morum* that both the heterologous *hsp*70A/*rbc*S3 tandem promoter of *V. carteri* and the heterologous *HSP70A* promoter of *C. reinhardtii* work in this species. Three out of the four constructs clearly lead to functional expression of the heterologous Gluc luciferase and the heterologous aminoglycoside 3′-phosphotransferase VIII (AphVIII) enzymes. It is unclear why transformants produced with pPsaD-GLuc (PanTJ…) didn’t produce significant luciferase activity.

The heterologous heat shock promoters retained their inducibility by heat when expressed in *P. morum*. The expression was up to 555 times higher in heat-shocked versus non-heat-shocked cultures.

## Conclusion

The introduction of foreign genes into the nuclear genome of *P. morum* by particle bombardment is now feasible. Both the heterologous *hsp*70A/*rbc*S3 tandem promoter of *V. carteri* and the heterologous *HSP70A* promoter of *C. reinhardtii* drive the expression of heterologous genes in *P. morum.* Both promoters retained their heat-inducibility in *P. morum*. Furthermore, the heterologous *aph*VIII gene of *S. rimosus* was established as a selectable marker and the *gluc* luciferase gene was found to be a useful reporter gene in *P. morum*. It is thus to be emphasized that genetic engineering of a species is possible even without the availability of any endogenous genes and promoters.

## Methods

### Strains and culture conditions

The wild-type *P. morum* strain SAG 32.96 was obtained from the Culture Collection of Algae at Goettingen University (SAG), Germany. Cultures were maintained in Jaworski medium (JM) [47] at 29°C in an 8 h dark/16 h light (~10,000 lux) cycle [11,20]. Cultures were grown in 10 ml glass tubes, 50 or 300 ml Erlenmeyer flasks, or 1,000 ml Fernbach flasks. The glass tubes had caps that allow for gas exchange, and Erlenmeyer and Fernbach flasks were aerated via Pasteur pipettes with approximately 50 cm^3^ sterile air/min [11,20]. Transgenic strains that express the *aph*VIII gene were grown in JM in the presence of 1 μg paromomycin/ml (paromomycin sulfate, Sigma-Aldrich, St. Louis, MO).

### Transformation vectors

The pPmr3 plasmid (Figure 3A) contains the 0.8 kb coding region of the *S. rimosus aphVIII* selectable marker gene, a *V. carteri hsp*70A-*rbc*S3 tandem promoter (0.5 kb of *V. carteri hsp*70A and 0.27 kb of *V. carteri rbc*S3 sequences), the 3′ UTR from the *V. carteri rbc*S3 gene (0.53 kb of downstream sequence), and the pBluescript II vector backbone [GenBank: AY429514] [31]. The total size of the pPmr3 plasmid is 5.1 kb.

The pPsaD-GLuc plasmid (Figure 3B) contains the 0.57 kb coding region of the luciferase (*gluc*) gene from *G. princeps*, which was engineered to match the codon usage in *C. reinhardtii*, 0.8 kb of upstream sequence, which includes the *C. reinhardtii PSAD* promoter, the 3′-UTR of the *C. reinhardtii psaD* gene (0.56 kb of downstream sequence), and the pBluescript vector backbone [27,36] [GenBank: EU372000, AF335592]. The pPsaD-GLuc plasmid is 5.0 kb in size.

The pHsp70A-GLuc plasmid (Figure 3C) contains the 0.57 kb coding region of the luciferase (*gluc*) gene from *G. princeps* (codon-optimized for *C. reinhardtii*) [27] fused downstream of a 0.8 kb genomic DNA fragment that contains the first three exons and two introns of the *HSP70B* gene of *C. reinhardtii*; this fragment of the *HSP70B* gene contains the sequence coding for the chloroplast transit peptide of HSP70B. The hybrid *HSP70B*/*gluc* gene is driven by the *C. reinhardtii HSP70A* promoter (0.26 kb of upstream sequence) and the 3′ UTR comes from the *C. reinhardtii RBCS2* gene (0.22 kb of downstream sequence) [27]. The total size of the pHsp70A-GLuc plasmid is 4.9 kb.

The pHRLucP plasmid (Figure 3D) contains the 0.57 kb coding region of the luciferase (*gluc*) gene of *G. princeps*, which was engineered to match the codon usage in *C. reinhardtii*, the *V. carteri hsp*70A-*rbc*S3 tandem promoter (0.5 kb of *V. carteri hsp*70A and 0.27 kb of *V. carteri rbc*S3 sequences), the *C. reinhardtii PSAD* 3′ UTR (0.55 kb of downstream sequence), and the pBluescript II vector backbone [11,27,36]. The pHRLucP plasmid is 5.0 kb in size.

### Preparation of plasmid DNA

Plasmid DNA was purified using the Nucleospin® Plasmid Kit according to the manufacturers’ instructions (Macherey-Nagel, Düren, Germany).

### Coating of microprojectiles

Gold microprojectiles (0.6 μm in diameter, Bio-Rad, Hercules, CA) were coated with the required plasmids for biolistic transformation as previously described [11,20]. The DNA-coated microprojectiles were resuspended in 60 μl of ethanol and kept at 4°C; these microprojectiles were used within 3 h of preparation.

### Determination of cell concentration

In *P. morum* the number of cells per colony varies. Therefore, we refer to “cells/ml” rather than “colonies/ml”. The concentration of cells was determined using a hemacytometer with Neubauer ruling.

### Stable nuclear transformation by particle bombardment

The stable transformation of *P. morum* was performed using a Biolistic® PDS-1000/He (Bio-Rad) particle gun. To this end, a logarithmically growing *P. morum* culture (150 ml) at a cell concentration of ~7 × 10^4^ cells/ml was harvested by centrifugation (1,000 g, 5 min, swing-out rotor) and resuspended in 6 ml of JM. One milliliter of the suspension was evenly spread on a cellulose acetate membrane filter and excess liquid was removed as previously described [11,20]. One-sixth of the DNA-coated microprojectiles were evenly spread on a macrocarrier (Bio-Rad) that was placed in a macrocarrier holder (Bio-Rad). The transformation procedure was as previously described [11,20] and the most successful combination of parameters is shown in Table 1. After the bombardment step, the algae were washed off from the membrane filter with JM. The procedure was repeated five times and the algae from six rounds of bombardment were pooled and then evenly distributed among twelve 50 ml Erlenmeyer flasks containing a volume of ~40 ml of JM. The bombarded colonies were incubated under standard conditions for 24 h to allow for regeneration and expression of the transgenes. Then, 2 μg of paromomycin/ml was added. The large fraction of non-transformed cells died within 48 h and the medium clarified. After 10–14 days of incubation in the presence of antibiotic, re-greening of flasks indicated the initial presence and reproduction of at least one paromomycin-resistant *P. morum* transformant that led to a population of transformants. No more than one transformant per flask was analyzed [11,20].

### Re-isolation of transformants

Transformants were re-isolated to ensure uniform genetic conditions for all further analyses. For this purpose, a serial dilution of an exponentially growing *P. morum* culture was performed in a Terasaki plate (Nunc™ MicroWell™ MiniTrays; Thermo Fisher Scientific, Langenselbold, Germany) [11,20]. Each well of the Terasaki plate was filled with 10 μl of JM. A single *P. morum* colony was finally transferred into a standard glass tube with JM, containing 1 μg of paromomycin/ml. Further incubation was under standard conditions.

### Paromomycin-resistance assay

Cells of transformed or wild-type *P. morum* strains were transferred into the wells of a 24-well culture plate (Sarstedt, Nümbrecht, Germany) with a wide range of paromomycin concentrations from 0 to 100 μg/ml in 1 ml JM (concentrations are given in Figure 2). At the beginning of the assay, each well contained approximately 4,000 cells, which corresponds to 250–300 colonies. After incubation under standard conditions for 12 days, the wells were analyzed for viable green cells or white remains of lysed cells.

### Primer design

Oligonucleotide primers were designed using the primer analysis software Oligo 6 (Molecular Biology Insights, Cascade, CO), DNASIS™ (version 7.00; Hitachi Software Engineering, San Francisco, CA), and Primer Express® (Applied Biosystems, Foster City, CA).

### Isolation of genomic DNA

Ten milliliters of a logarithmically growing *P. morum* culture with a density of 6 × 10^6^ cells/ml was harvested by centrifugation (3,500 g for 10 min). The resulting pellet, which had a wet weight of ~80 mg, was washed with water and resuspended in 40 μl water. Isolation of genomic DNA was as previously described [11,20].

### Genomic PCR

PCR reactions with genomic DNA as a template were carried out in a total volume of 50 μl that contained ~100 ng of genomic DNA, 300 nM of each primer, 0.2 mM dNTP, 1.5 mM MgCl_2_, and 2.6 units of Expand High Fidelity enzyme mix in 1× Expand High Fidelity buffer (Roche Applied Science, Basel, Switzerland). The PCR reactions were performed on a T3 Thermocycler PCR system (Biometra, Göttingen, Germany) using the following conditions: 40 cycles of 94°C for 20 s, 55°C for 30 s, and 72°C for 45 s and a final extension was at 72°C for 10 min. The PCR products were cloned and sequenced (Eurofins, Ebersberg, Germany).

### Isolation of total RNA

Total RNA was extracted from ~100 mg of concentrated, frozen *P. morum* algae using 1 ml of phenol-based TRI Reagent (Sigma-Aldrich, St. Louis, MO) and 300 μl trichloromethane. RNA precipitation and RNA purification was as previously described [11,20].

### Reverse Transcription (RT)-PCR

First strand cDNA synthesis was carried out using 1 μg of total RNA and Moloney murine leukemia virus (MMLV) reverse transcriptase lacking ribonuclease H activity (H minus) according to the manufacturer’s instructions (Promega). The subsequent PCR step was performed using the Expand High Fidelity PCR system (Roche Applied Science, Basel, Switzerland), a T3 Thermocycler PCR system (Biometra), and the following cycling conditions: 40 cycles of 94°C for 20 s, 55°C for 30 s, and 72°C for 45 s and a final extension at 72°C for 10 min. The RT-PCR products were cloned and sequenced.

### Induction of luciferase activity

Preliminary tests showed that the shift of a *P. morum* culture (3–6 × 10^5^ cells/ml) from 29°C to 39°C for 1 h, followed by a recovery phase at 29°C for 15 min, leads to the strongest induction of luciferase activity in the temperature range investigated (27°C to 51°C).

### Luciferase assays

For assays on light-sensitive films, *P. morum* cultures with a cell density of 3–6 × 10^5^ cells/ml were divided into aliquots of 50 ml. One aliquot was incubated at the optimal temperature for heat stress-induced expression of luciferase (39°C) for 1 h, and another aliquot was incubated at 29°C as a reference control. Further treatment and execution of the luciferase enzyme assay was as previously described [11,20]. The final exposure to a chemiluminescence sensitive film (Retina XBA; Fotochemische Werke) was for 2 h at 20°C [11,20,35].

The quantification of luciferase activity was conducted as previously described [20,27] using a MiniLumat LB9506 luminometer (Berthold, Bad Wildbad, Germany). For this purpose, aliquots of a *P. morum* culture (3–6 × 10^5^ cells/ml) were heat-stressed at 39°C or kept at 29°C (control), respectively. Further treatment was as previously described [20]. Luminescence was recorded as relative light units (rlu). Induction factors were calculated by comparison of samples from heat-shocked versus non-heat-shocked cultures.

### In-gel activity assay

One liter of logarithmically growing, heat-stressed or untreated cultures of transformed or wild-type *P. morum* cultures with cell densities of 4–7 × 10^5^ cells/ml were harvested by filtration on a 10-μm mesh nylon screen. Cells passing the screen were harvested by centrifugation (4,000 g, 5 min) and added to cells collected on the screen. Cells lysates were produced as previously described [11]. The protein concentration of the cell extracts was determined photometrically using the Bio-Rad Protein Assay Dye Reagent (Bio-Rad). For gel electrophoresis, 50 μg of total protein was loaded onto a standard SDS-polyacrylamide gel. The gel electrophoresis, the in-gel renaturation, the execution of the luciferase enzyme assay and the light detection on a chemiluminescence sensitive film was performed as previously described [11].

## Abbreviations

*aph*VIII: *S. rimosus* aminoglycoside 3′-phosphotransferase VIII gene; GFP: Green fluorescent protein; *gluc*: *G. princeps* luciferase gene (adapted to *C. reinhardtii* nuclear codon usage); *hsp*70A: Heat shock protein 70A gene; JM: Jaworski medium; kDa: Kilodalton; MMLV: Moloney murine leukemia virus; PCR: Polymerase chain reaction; *PSAD*: Abundant protein of photosystem I complex gene; *rbc*S3: Ribulose bisphosphate carboxylase small chain gene 3; rlu: Relative light units; RT: Reverse transcription; SDS: Sodium dodecyl sulfate; UTR: Untranslated region.

## Competing interests

The authors declare that they have no competing interests.

## Authors’ contributions

KL conducted the experiments, analyzed the data, and wrote the first version of the manuscript with advice and guidance from AH. AH (corresponding author) conceived and coordinated the study, critically evaluated the data, and finalized the manuscript. Both authors read and approved the final manuscript.

## Supplementary Material

Additional file 1 Influence of different parameters on transformation efficiency. Click here for file
