# Supplementary material for: Stable nuclear transformation of Pandorina morum
Source: BMC Biotechnol. 2014 Jul 17;14:65. doi: 10.1186/1472-6750-14-65 (PMC4115218; doi:10.1186/1472-6750-14-65)
Supplement: Additional file 1 — Influence of different parameters on transformation efficiency. [file 1472-6750-14-65-S1.pdf]

Influence of different parameters on transformation efficiency

Parameter and parameter specification	Efficiency
**Transformation method**	
glassbeads (untreated cells)	-
glassbeads (pretreated with protease)	-
particle gun (homemade, without vacuum chamber)	+
particle gun (PDS-1000/He biolistic device, with vacuum chamber)	+ + + + +
**Material of microprojectiles**	
gold	+ + + + +
tungsten	+
**Size of microprojectiles**	
0.6 µm in diameter	+ + + + +
1.0 µm in diameter	+ + +
1.6 µm in diameter	+
**Selectable marker plasmid**	
pPmr3	+ + + + +
promoterless *aphVIII*	(+)
**Coating of microprojectiles**	
plasmid-DNA/microcarrier/NaAc/EtOH-precipitation	+ +
plasmid-DNA/microcarrier/$CaCl_2$/spermidine/EtOH-precipitation	+ + + + +
**Target cells**	
resuspended in as less liquid as possible; spread in an empty Petri dish	-
immobilized on moist filter paper; more or less free of liquid	+ +
immobilized on cellulose acetate membrane filter; almost free of liquid	+ + + + +

**Burst pressure of rupture disks**	
650 psi	-
900 psi	-
1100 psi	+ + +
1350 psi	+ + + + +
1550 psi	+ +
**Rupture disk-macrocarrier distance**	
7 mm	+ + + + +
16 mm	+ +
**Macrocarrier-stopping screen distance**	
8 mm	+ + + + +
12 mm	+ + + +
18 mm	+ + +
**Stopping screen-target cell distance**	
6 cm	+ + + + +
11 cm	+
18 cm	-
**Chamber evacuation**	
(almost) no evacuation	-
15 inch Hg	+ +
27 inch Hg	+ + + + +
**Cultivation after particle bombardment**	
on agar plates	-
in liquid medium	+ + + + +

Within each subgroup, the condition that gave the largest number of transformants was set to "+ + + + +"; there is a "-" when no transformants were recovered. The data allow just a qualitative estimation, because a) they were obtained under varying other conditions in the course of optimization, b) poor results disqualified a condition without rechecking, and c) some of the transformants might be false-positives as only a few were analyzed in detail.
